# Lung Compliance and Chronic Obstructive Pulmonary Disease

**DOI:** 10.1155/2012/542769

**Published:** 2012-10-22

**Authors:** D. Papandrinopoulou, V. Tzouda, G. Tsoukalas

**Affiliations:** 4th Department of Pulmonology, Athens Hospital for Chest Diseases “Sotiria”, 152 Mesogeion Street, Athens 11527, Greece

## Abstract

Chronic obstructive pulmonary disease, namely, pulmonary emphysema and chronic bronchitis, is a chronic inflammatory response of the airways to noxious particles or gases, with resulting pathological and pathophysiological changes in the lung. The main pathophysiological aspects of the disease are airflow obstruction and hyperinflation. The mechanical properties of the respiratory system and its component parts are studied by determining the corresponding volume-pressure (*P-V*) relationships. The consequences of the inflammatory response on the lung structure and function are depicted on the volume-pressure relationships.

## 1. Introduction

Chronic obstructive pulmonary disease (COPD), a common preventable and treatable disease, is characterized by persistent airflow limitation that is usually progressive and associated with an enhanced chronic inflammatory response in the airways and the lung to noxious particles or gases [1]. Airway limitation is attributed to three different mechanisms: (1) partial block of the lumen (e.g., due to excessive mucous production forming semisolid plugs), (2) thickening of the airway wall, which occurs because of edema or muscle hypertrophy, and (3) abnormality of the tissue surrounding the airways (destruction of the parenchyma and narrowing of the airway due to loss of radial traction). Both entities of COPD, namely, chronic bronchitis and emphysema, are characterized by the former mechanisms. Chronic bronchitis is characterized by partial block of the lumen and airway wall thickening, while emphysema by radial traction loss [2].

The most common risk factor for COPD globally is cigarette smoke. Cigarette smokers show a higher prevalence of respiratory symptoms and lung function abnormalities than nonsmokers. As a consequence, the annual rate of decline in FEV_1_ is higher than the expected FEV_1_ decline with decreasing age. Passive exposure to smoke is also an important aspect of the disease. We should take under consideration the occupational exposures, including organic and inorganic dusts, chemical agents and fumes, and of course the indoor pollution from the biomass during cooking and heating in poorly ventilated houses [3–5]. 

## 2. COPD Pathology 

Pathological changes are found in large and small airways, in the parenchyma and the pulmonary vasculature, resulting from repeated injury and repair. The inflammatory response may be genetically determined, or may be caused by noxious particles, such as cigarette smoke. However, some patients develop COPD without exposure to cigarette smoke. The oxidative stress and the imbalance between proteases and antiproteases, which have a role in protection of connective tissue from breaking down, amplify the inflammatory response of the disease. The inflammatory response is aggravated by the inflammatory cells CD8+ cytotoxic Tc1 lymphocytes and the inflammatory mediators (chemotactic factors, proinflammatory cytokines and growth factors) [6]. 

A scenario which is under experimental exploration exposes an attractive model for initiation of inflammation, comprising oxidative DNA damage of LEBCs and host immune response. According to that, noxious particles induce oxidative DNA damage of the lung epithelial barrier cells (LEBCs), and the acquired mutations are expressed at the microsatellite DNA level of LEBCs. The altered LEBCs are recognized by dendritic cells (DCs) as “nonself” DCs travel with the new information to the lymph nodes, presenting it to the naïve T lymphocytes, and after that a predominant CD8+ cytotoxic T-lymphocyte proliferation occurs. The CD8+ T lymphocytes release perforin and granzymes and attract the altered LEBCs, activating cell death cascades [7].

## 3. Pathophysiological Aspects in COPD

### 3.1. Airflow Obstruction

Airflow during exhalation is the result of the balance between the elastic recoil of the lungs promoting flow and resistance of the airways that limits flow. The factors that lead to the obstruction of the lumen and the increased resistance, with the consequence of flow limitation, are the presence of secretions, the increased tone of bronchial smooth muscle, the hypertrophy of submucosal glands, and the protrusion towards the internal part of the lumen of the dorsal part of trachea during expiration. These factors are involved in airflow obstruction due to the great difference between intraluminal pressure and the pressure in the surroundings. The loss of elastic recoil concerning the wall of the small airways, due to the reduction of elastic tissue in the pulmonary parenchyma, is an evident mainly in emphysema. The absence of cartilage in the wall of the small peripheral airways contributes further more to the loss of the elastic recoil [8]. Patients with COPD are said to be flow limited when the expiratory flow that they generate during tidal respiration represents the maximal possible flow that they can generate at that volume. In flow-limited patients, the time available for lung emptying (expiratory time) during spontaneous breathing is often insufficient to allow end expiratory lung volume (EELV) to decline to its natural relaxation volume. This leads to lung hyperinflation [9].

### 3.2. Hyperinflation

The loss of the elastic recoil, especially in the case of emphysema, the fact that the COPD patient breaths in “a higher level” which means that the functional tests show a functional residual capacity (FRC) which exceeds the predicted one, in order to maintain the airways open and the air trapping during premature closure are all aspects of lung hyperinflation [8].

In normal subjects, lung volume at end expiration approximates the relaxation volume of the respiratory system. However, in patients with airflow obstruction, the end-expiratory lung volume may exceed the predicted FRC. Indeed, lung emptying is slowed, and expiration is interrupted by the next inspiratory effort, before the patient has reached the static equilibrium volume [11]. This is termed dynamic hyperinflation and is affected by VT, expiratory time, resistance, and compliance. It is also called intrinsic positive end-expiratory pressure (auto-PEEP) and was firstly described by Bergman in 1972 [12] and Johnson et al. in 1975 and represents the positive intrapulmonary pressure at the end of expiration [13]. The presence of auto-PEEP means that the inspiratory muscles must firstly overcome the combined inward recoil of the lung and the chest wall before inspiratory flow can be initiated. Thus, auto-PEEP acts essentially as an inspiratory threshold load and has been measured to be as much as 6–9 cmH_2_O during quiet breathing at rest in clinically stable but hyperinflated COPD patients [14, 15]. 

During severe airflow obstruction episodes, increased expiratory efforts simply raise alveolar pressure without improving expiratory airflow. When tidal volume (VT) is increased or the expiratory time is short because of a high respiratory rate, the lung cannot deflate to its usual resting equilibrium between breaths. This raise in alveolar pressure and lung volume results in several events which affect the dynamic status of the lung. Breathing takes place near the total lung capacity [16]. Tidal breathing during an exacerbation in a patient with COPD may be shifted upwards close to the total lung capacity as a consequence of dynamic hyperinflation [17].

Hyperinflation has detrimental effects on the function of diaphragm that increase the work of breathing. First of all, the diaphragm is displaced into a flattened position which results in the decrease of the zone of apposition between the diaphragm and the abdominal wall. Secondly, the muscle fibers of the flattened diaphragm are shorter and are less capable of generating inspiratory pressures that will overwhelm the transpulmonary pressure [18]. The positive pressure within the hyperinflated lung raises the mean intrathoracic pressure and causes the inspiratory muscles to operate at a higher than resting lung volume [19]. The sarcomere length of the diaphragm in COPD patients is shorter and indirectly proportional to the TLC and the RV. This adjustment improves the capability of the diaphragm to generate force in “higher lung volumes.” The ideal length of inspiratory muscles during relaxation is considered to be near the level of residual volume (RV). In COPD patients, however, because of hyperinflation, the length of the muscle fibers is even shorter. Furthermore, an increase in the relative proportion of the type I fibers which are slow twitch and fatigue resistant [20, 21] and an increase in mitochondrial concentration and efficiency of the electron transport chain which improves oxidative capacity are other structural adaptations to chronic intrinsic mechanical loading [22]. As a result, the developing force produced by the muscles is even more reduced on the expense of a considerable mechanical disadvantage which further impairs respiratory muscle function rendering this way the diaphragm more weak [18]. 

The ventilatory muscles partially adapt to chronic hyperinflation to preserve their force generating capacity during resting breathing. During COPD exacerbations, these adaptations can become overwhelmed [20–22]. The already burdened inspiratory muscles become subject to increased elastic loading, which means that they require a greater effort for a given change of volume. Acute dynamic hyperinflation further shortens the inspiratory muscles, particularly the diaphragm, and causes functional muscle weakness [23–25]. Exposure to oxidative stress and local activation of proteases may also result in diaphragmatic injury during periods of increased inspiratory loading and result in inspiratory muscle dysfunction [26, 27]. The net effect of this increased loading and functional weakness of the inspiratory muscles is that the effort required for tidal inspiration represents a relatively high fraction of the maximal possible effort that the patient can develop at that lung volume [28].

## 4. Lung Compliance 

The respiratory system owns its elastic property to the function of the respiratory muscles, which supply the whole system with the necessary pressure difference so that air moves into the airways. The static mechanical properties of the respiratory system and its component parts are studied by determining the corresponding pressure-volume (*P-V*) relationships. The *P-V* curves that are obtained as volume is changed in progressive steps from residual volume (RV) to total lung capacity (TLC) and back again are loops. The elastic properties of the lung and chest wall as well as the changes in lung units between inflation and deflation are responsible for the presence of these loops [16]. The lung elasticity is depicted by the static volume-pressure curve. The fact that the *P-V* curve is nonlinear practically means that as lung volume increases, the elastic elements approach their limits of distensibility [29]. 

**Figure 1:**
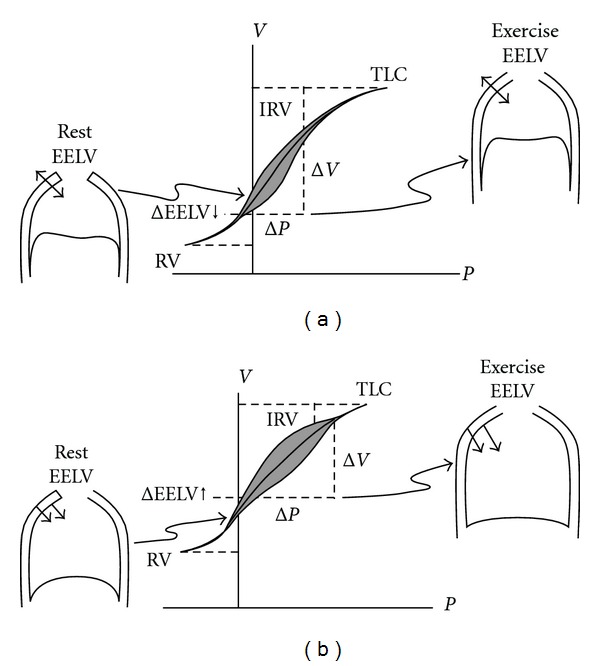
Pressure-Volume (*P-V*) relationships of the total respiratory system in (a) normal and (b) chronic obstructive pulmonary disease (COPD). Tidal *P-V* curves during rest and during exercise are shown. In COPD individuals, there is a resetting of the respiratory system's relaxation volume to a higher level than in the healthy individuals. Hyperinflation in COPD leads to increased EELV, RV and a corresponding reduction in IRV, in comparison to normal condition. In contrast to normal lung, the combined recoil pressure of the lungs and chest wall in hyperinflation is inwardly directed during rest and during exercise. This results in inspiratory threshold load on the inspiratory muscles with consequential decrease in the zone of apposition (shown in *P-V* curve (b) during rest and exercise). EELV: end-expiratory lung volume, RV: residual volume, IRV: inspiratory reserve volume, TLC: total lung capacity. From O'Donnell and Laveneziana [30].

In COPD, because of the resting and dynamic hyperinflation which is equal to a further increase in the end-expiratory lung volume (EELV), the exercise tidal volume (VT) encroaches on the upper alinear extreme of the respiratory system's *P-V* curve where there is increased elastic loading. In COPD, the ability to further expand VT is reduced, so inspiratory reserve volume (IRV) is reduced. In contrast to health, the combined recoil pressure of the lungs and chest wall in hyperinflated patients with COPD is inwardly directed during both rest and exercise. This results in an inspiratory threshold load on the respiratory muscles (Figure 1) [30]. Intrapulmonary pressures do not return to zero, representing this way the auto-PEEP which imposes extra load to the inspiratory muscles. During the subsequent respiratory cycle, auto-PEEP must be overcome in order to generate inspiratory flow [17]. 

The static compliance (*C*) of the lung is the change in lung volume per unit change in the transpulmonary pressure; that is, the pressure difference between the interior of the alveoli and the pleural surface of the lungs required to affect a given change in the volume of air in the lungs:

(1) C=ΔVLΔ(PA−PPl),

where *C* is the compliance, Δ*V*_*L*_ is the change in lung volume, and Δ(*P*_*A*_ − *P*_Pl_) is the change in the transpulmonary pressure.

Strictly speaking, the transpulmonary pressure is equal to the pressure in trachea minus the intrapleural pressure. Thus, it is the pressure difference across the whole lung. However, the pressure in the alveoli is the same as the pressure in the airways (including the trachea) at the beginning or at the end of each normal breath. That is, the end-expiratory or end-inspiratory alveolar pressure is 0 cm H_2_O. Therefore, at the beginning or at the end of each lung inflation, alveolar distending pressure can be referred to as the transpulmonary pressure [31].

**Figure 2:**
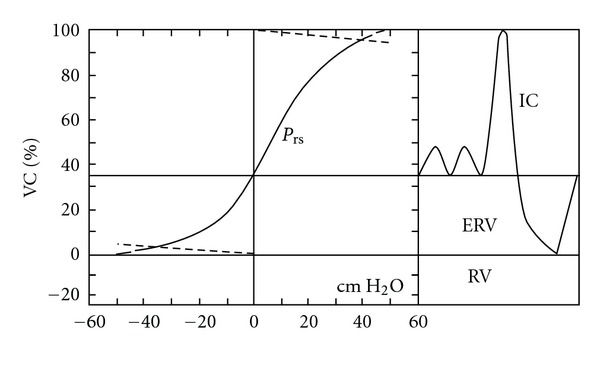
Quasistatic *P-V* curve of the respiratory system, with a spirogram showing subdivisions of lung volume. ERV: expiratory reserve volume, IC: inspiratory capacity, VC: vital capacity. Adapted from Agostini and Hyatt [32].

The volume that corresponds to zero pressure (0 cm H_2_O) is the resting volume of the respiratory system, where the pressure at the level of the mouth is 0 cm H_2_O. This is the level of functional residual capacity (FRC) where there is no airflow in the airways and the pressure at the level of the mouth when performing spirometric studies equals the airway and the alveolar pressure (0 cm H_2_O). In the *P-V* curve, the horizontal distance from the point 0 represents the elastic pressure of the whole respiratory system (*P*_pl_) which is negative below the FRC and positive above the FRC level (Figure 2) [32]. The pressure difference between the pressure at the level of the mouth and the atmospheric pressure represents the pressure needed to expand the respiratory system during the respiratory cycle.

The looped *P-V* curve practically means that as lung volume increases, the elastic elements approach their limits of distensibility and a given change in transpulmonary pressure produces smaller and smaller increases in lung volume. As a result, the compliance of the lung is the least at high lung volumes and greatest as the residual volume (RV) is approached [18]. A lung of high compliance expands to a greater extend than one of lower compliance when both are exposed to the same increase in transpulmonary pressure [10]. Hyperinflation and tidal breathing towards the total lung capacity force the respiratory system to operate on the flatter part of the compliance curve where progressive pressure increases generate smaller incremental volume changes [17].

The lung compliance is normally measured as static, when the lungs are stationary. The distensibility estimated during normal tidal breathing from measurements of lung volume and esophageal pressure made at the end of inspiration and expiration when the lungs are apparently stationary has an index as a result, which is called dynamic compliance. In subjects with healthy lungs, the two measurements yield similar results [10]. 

Since the pressure-volume curve of the lung requires estimation of the pressures in the airways and around the lung, this can be obtained by measurement of the esophageal pressure. A small balloon on the end of the catheter is passed down through the nose or mouth, and the difference between the mouth and the esophageal pressures is recorded as the patient exhales in steps of 1 liter from the total lung capacity (TLC) to RV [2].

Some factors that influence the static lung compliance (including emphysema) are listed in Table 1. 

## 5. *P-V* Curve and Emphysema 

Most of the early studies describing the *P-V* relationship in COPD were done to diagnose and establish the severity of emphysema. However CT was more convenient as a method. Most of the early study were done on spontaneously breathing COPD subjects [33].

Gibson et al. found that the *k* factor was increased in COPD patients. The *k* factor describes the concavity of the exponential fit and is independent of lung volume. Therefore, an increase in *k* means the curve has concavity toward the pressure axis, without regard to its position [34]. This was confirmed by the study of Greaves and Colebatch who studies normal and emphysematous lungs. They found that when emphysema was present, *k* was increased by more than two standard deviations above the mean predicted value for age. They also found a direct relationship between *k* and mean alveolar size [35]. Osborne et al. studied the relationship between *k* and mean alveolar size in emphysematous undergoing lung resection. They found that *k* correlates with severity of COPD until the contribution of large air spaces to the shape of the curve was lost due to airway closure [36].

Compliance is increased in obstructive lung disease like pulmonary emphysema, less in asthma and at a minor degree in chronic bronchitis. In emphysema, the elastic recoil is decreased and the *P-V* curve is shifted up and left. This is due to the loss of elastic tissue as a result of alveolar wall destruction. In chronic bronchitis without emphysema, however, the *P-V* curve may be normal since the parenchyma is minimally affected.

In practice the measurement of compliance and its result has limited clinical value. As mentioned previously, the resulting curve is non linear and the value of compliance is measured according to the change in pressure. The measurement above the level of FRC, approaching the TLC, shows lower values of compliance and increased lung stiffness due to the collagen fibers in the lung parenchyma, which influence the lung distension in high volumes. Thus, the measurement of compliance should be carried out close to the FRC level, otherwise, close to the TLC and RV levels, the results have limited clinical value [37].

The natural history of the development of lung hyperinflation in COPD patients according to clinical experience indicates that it is an insidious process that occurs over decades. It would appear that RV is the first volume component to increase, reflecting increased airway closure. EELV increases thereafter, reflecting the effects of EFL and alteration in lung mechanics, and eventually TLC increases as lung compliance increases. However, it is likely that the time course of change in the various volume compartments is highly variable among patients [30, 38].

The *P-V* curve shows different configuration during the respiratory cycle, that is, during inspiration and expiration. This phenomenon is called hysteresis and is a property of elastic structures. The difference in configuration occurs due to the fact that close to the RV (small lung volumes) further pressure is required during inspiration to open the small distal airways. In greater lung volumes, the phenomenon of hysteresis is possibly attributed to the resistance of elastic fibers in the parenchyma [37].

## 6. Conclusion

The static and dynamic studies of the lung in chronic obstructive pulmonary disease differ according to the pathological aspects of the disease. The loss of elastic recoil of the lung affects the pressure difference between the interior of the alveoli and the pleural surface of the lungs, that is, the transpulmonary pressure. As a result, a lung of high compliance, like the emphysematous lung, expands to a greater extent than the one of low compliance, when both are exposed to the same increase in transpulmonary pressure.

## Figures and Tables

**Table 1:** 

Aspect	Low compliance	High compliance
Lung of normal structure	Small person	Large person

Lung surfactant	Respiratory distress syndrome. Surfactant protein B deficiency	

Fibrous stroma	Disorders of lung parenchyma	Age, emphysema, and semicarbazide

Visceral pleural	Thickening secondary to TB, Asbestos exposure, and haemothorax	

Tone in muscle of alveolar ducts	Histamine	Bronchodilator drugs
	Serotonin, hypoxia	

Pulmonary blood volume	Mitral stenosis	isocapnoeic hypoxia
	Left ventricular failure	Pulmonary stenosis

Source: [10].

## References

[1] Global strategy for the diagnosis, management, and prevention of chronic obstructive pulmonary disease (GOLD). http://www.gold.copd.org/.

[2] West JB (2007). *Pulmonary Pathophyiology: The Essentials*.

[3] Trupin L, Earnest G, San Pedro M (2003). The occupational burden of chronic obstructive pulmonary disease. *European Respiratory Journal*.

[4] Matheson MC, Benke G, Raven J (2005). Biological dust exposure in the workplace is a risk factor for chronic obstructive pulmonary disease. *Thorax*.

[5] Ezzati M (2005). Indoor air pollution and health in developing countries. *The Lancet*.

[6] Cosio MG, Saetta M, Agusti A (2009). Immunologic aspects of chronic obstructive pulmonary disease. *The New England Journal of Medicine*.

[7] Tzortzaki EG, Siafakas NM (2009). A hypothesis for the initiation of COPD. *European Respiratory Journal*.

[8] Macklem PT (1973). The pathophysiology of chronic bronchitis and emphysema. *Medical Clinics of North America*.

[9] O’Donnell DE, Webb KA, Calverley PMA, MacNee W, Pride NB, Rennard SI (2003). Exercise. *Chronic Obstructive Pulmonary Disease*.

[10] Cotes JE, Chinn DJ, Miller MR *Lung Function*.

[11] Gottfried SB, Rossi A, Milic-Emili J (1986). Dynamic hyperinflation, intrinsic PEEP, and the mechanically ventilated patient. *Intensive and Critical Care Digestion*.

[12] Bergman NA (1972). Intrapulmonary gas trapping during mechanical ventilation at rapid frequencies. *Anesthesiology*.

[13] Jonson B, Nordstrom L, Olsson SG, Akerback D (1975). Monitoring of ventilation and lung mechanics during automatic ventilation: a new device. *Bulletin de Physio-Pathologie Respiratoire*.

[14] Pare PD, Brooks LA, Bates J (1982). Exponential analysis of the lung pressure-volume curve as a predictor of pulmonary emphysema. *The American Review of Respiratory Disease*.

[15] Haluszka J, Chartrand DA, Grassino AE, Milic-Emili J (1990). Intrinsic PEEP and arterial PCO_2_ in stable patients with chronic obstructive pulmonary disease. *The American Review of Respiratory Disease*.

[16] Blanch L, Bernabé F, Lucangelo U (2005). Measurement of air trapping, intrinsic positive end-expiratory pressure, and dynamic hyperinflation in mechanically ventilated patients. *Respiratory Care*.

[17] O’ Donnell DE, Parker CM (2006). COPD exacerbations *·* 3: pathophysiology. *Thorax*.

[18] Fishman AP, Elias J, Fishman JA (1997). *Fishman’s Pulmonary Diseases and Disorders*.

[19] Dhand R (2005). Ventilator graphics and respiratory mechanics in the patient with obstructive lung disease. *Respiratory Care*.

[20] Levine S, Kaiser L, Leferovich J, Tikunov B (1997). Cellular adaptations in the diaphragm in chronic obstructive pulmonary disease. *The New England Journal of Medicine*.

[21] Mercadier JJ, Schwartz K, Schiaffino S (1998). Myosin heavy chain gene expression changes in the diaphragm of patients with chronic lung hyperinflation. *The American Journal of Physiology*.

[22] Orozco-Levi M, Gea J, Lloreta JL (1999). Subcellular adaptation of the human diaphragm in chronic obstructive pulmonary disease. *European Respiratory Journal*.

[23] Orozco-Levi M (2003). Structure and function of the respiratory muscles in patients with COPD: impairment or adaptation?. *European Respiratory Journal*.

[24] Laghi F, Tobin MJ (2003). Disorders of the respiratory muscles. *The American Journal of Respiratory and Critical Care Medicine*.

[25] Polkey MI, Kyroussis D, Hamnegard CH (1996). Diaphragm strength in chronic obstructive pulmonary disease. *The American Journal of Respiratory and Critical Care Medicine*.

[26] Similowski T, Yan S, Gauthier AP, Macklem PT, Bellemare F (1991). Contractile properties of the human diaphragm during chronic hyperinflation. *The New England Journal of Medicine*.

[27] Orozco-Levi M, Gea J, Lloreta JL (1999). Subcellular adaptation of the human diaphragm in chronic obstructive pulmonary disease. *European Respiratory Journal*.

[28] Chen Z, Eldridge FL, Wagner PG (1992). Respiratory-associated thalamic activity is related to level of respiratory drive. *Respiration Physiology*.

[29] D’ Angelo E, Emili JM, Hamid Q, Shannon J, Martin J (2005). Statics of the respiratory system. *Physiologic Basis of Respiratory Disease*.

[30] O’ Donnell DE, Laveneziana P (2006). Physiology and consequences of lung hyperinflation in COPD. *European Respiratory Review*.

[31] Levitzky MG (2000). *Pulmonary Physiology*.

[32] Agostini E, Hyatt R, Macklem PT, Mead J (1986). Static behavior of the respiratory system. *Handbook of Physiology*.

[33] Harris RS (2005). Pressure-volume curves of the respiratory system. *Respiratory Care*.

[34] Gibson GJ, Pride NB, Davis J, Schroter RC (1979). Exponential description of the static pressure-volume curve of normal and diseased lungs. *The American Review of Respiratory Disease*.

[35] Greaves IA, Colebatch HJH (1980). Elastic behavior and structure of normal and emphysematous lungs post mortem. *The American Review of Respiratory Disease*.

[36] Osborne S, Hogg JC, Wright JL, Coppin C, Pare PD (1988). Exponential analysis of the pressure-volume curve: correlation with mean linear intercept and emphysema in human lungs. *The American Review of Respiratory Disease*.

[37] Murray JE (1986). *The Normal Lung-Ventilationc*.

[38] Leith DE, Brown R (1999). Human lung volumes and the mechanisms that set them. *European Respiratory Journal*.

